# Predicting prognosis for epithelial ovarian cancer patients receiving bevacizumab treatment with CT-based deep learning

**DOI:** 10.1038/s41698-024-00688-6

**Published:** 2024-09-13

**Authors:** Xiaoyu Huang, Yong Huang, Kexin Liu, Fenglin Zhang, Zhou Zhu, Kai Xu, Ping Li

**Affiliations:** 1 Department of Chinese Integrative Medicine Oncology, The First Affiliated Hospital of Anhui Medical University, Hefei, China; 2 Department of Medical Oncology, The Second People’s Hospital of Hefei, Hefei, China; 3 Scholl of Internet, Anhui university, Hefei, China; 4 Graduate School of Anhui University of Traditional Chinese Medicine, Hefei, China

**Keywords:** Ovarian cancer, Risk factors, Prognostic markers

## Abstract

Epithelial ovarian cancer (EOC) presents considerable difficulties in prognostication and treatment strategy development. Bevacizumab, an anti-angiogenic medication, has demonstrated potential in enhancing progression-free survival (PFS) in EOC patients. Nevertheless, the identification of individuals at elevated risk of disease progression following treatment remains a challenging task. This study was to develop and validate a deep learning (DL) model using retrospectively collected computed tomography (CT) plain scans of inoperable and recurrent EOC patients receiving bevacizumab treatment diagnosed between January 2013 and January 2024. A total of 525 patients from three different institutions were retrospectively included in the study and divided into training set (*N* = 400), internal test set (*N* = 97) and external test set (*N* = 28). The model’s performance was evaluated using Harrell’s C-index. Patients were categorized into high-risk and low-risk group based on a predetermined cutoff in the training set. Additionally, a multimodal model was evaluated, incorporating the risk score generated by the DL model and the pretreatment level of carbohydrate antigen 125 as input variables. The Net Reclassification Improvement (NRI) metric quantified the reclassification performance of our optimal model in comparison to the International Federation of Gynecology and Obstetrics (FIGO) staging model. The results indicated that DL model achieved a PFS predictive C-index of 0.73 in the internal test set and a C-index of 0.61 in the external test set, along with hazard ratios of 34.24 in the training set (95% CI: 21.7, 54.1; *P* < 0.001) and 8.16 in the internal test set (95% CI: 2.5, 26.8; *P* < 0.001). The multimodal model demonstrated a C-index of 0.76 in the internal test set and a C-index of 0.64 in the external test set. Comparative analysis against FIGO staging revealed an NRI of 0.06 (*P* < 0.001) for the multimodal model. The model presents opportunities for prognostic assessment, treatment strategizing, and ongoing patient monitoring.

## Introduction

Ovarian cancer is recognized as a highly lethal gynecologic malignancy, ranking sixth in terms of mortality rates among women^1^. Epithelial ovarian cancer (EOC) represents the most prevalent subtype, accounting for approximately 90% of all ovarian cancer cases. The standard treatment regimen for patients with EOC typically involves debulking surgery followed by platinum-based chemotherapy^2^. In the realm of clinical practice, some patients present with surgical contraindications upon initial diagnosis, rendering them ineligible for ovarian tumor debulking surgery. Furthermore, individuals who encounter recurrence following debulking surgery in conjunction with platinum-containing chemotherapy may be precluded from undergoing further tumor reduction surgery due to factors such as extensive tumor burden. In cases of postoperative recurrence and ineligibility for surgical intervention, the recommended course of treatment is platinum-based chemotherapy. However, a considerable number of patients experience relapse within three years of platinum-based chemotherapy^3^, with subsequent recurrences characterized by a diminishing progression-free survival (PFS)^4^. Despite the emergence of various treatment approaches in ovarian cancer, drug resistance has developed, leading to therapeutic refractoriness. Although bevacizumab treatment, which is a combination of the anti-angiogenic drug bevacizumab with platinum-based chemotherapy has demonstrated efficacy in extending PFS in certain patients, its utility is constrained by limitations^5,6^. The prognosis of EOC patients receiving bevacizumab treatment is influenced by factors such as disease stage, recurrence rate, and the development of drug resistance^7^. Due to the considerable expense, potential adverse effects, and notable variability in treatment effectiveness among individuals, it is imperative to develop a precise prognostic prediction model prior to initiating bevacizumab therapy.

The clinical management and prognostic evaluation of ovarian cancer are heavily reliant on the stage of the disease^8^. Ovarian cancer staging guidelines are established by the International Federation of Gynecology and Obstetrics (FIGO), providing a crucial framework for healthcare professionals to make prognostic assessments. It is important to note that patients with the same FIGO stage may experience varying survival rates. Carbohydrate antigen 125 (CA125) has become a prominent biomarker in ovarian cancer screening^9,10^, attracting significant attention and utilization in clinical practice. While it is valuable for evaluating response to chemotherapy and predicting prognosis, the clinical effectiveness of CA125 is a topic of debate^11^. Therefore, there is an urgent requirement to improve prognostic accuracy by integrating additional indicators.

Prior to establishing a treatment plan, ovarian cancer patients commonly undergo thorough clinical evaluations, including physical examinations, blood tests, and computed tomography (CT) scans. The amalgamation of information obtained from these various assessments plays a crucial role in predicting prognosis^12,13^. Nevertheless, traditional statistical approaches may face difficulties in analyzing the complex and extensive nature of multimodal data. Recent advancements in artificial intelligence have significantly enhanced the ability to analyze complex datasets^14^. Deep learning (DL) has emerged as a particularly promising approach within machine learning for the examination of multimodal data, eliminating the need for domain experts to manually extract or curate features^15^, as opposed to conventional machine learning techniques. It has the inherent ability to process raw data directly and independently generate necessary representations essential for pattern recognition, thus bypassing the explicit definition of rules or characteristics^16^. When provided with sufficient data points, deep learning has demonstrated superior performance compared to traditional radiological analyses^17^. Recent advancements in deep learning techniques have even resulted in achieving expertise comparable to experienced medical professionals in various medical image analysis tasks^18,19^.

This study involved the development and validation of a DL model, referred to as the risk score, utilizing preprocessed CT images of EOC tumors. The main objective was to predict the survival outcomes of EOC patients receiving bevacizumab treatment. Additionally, we investigated the potential enhancement of the model’s predictive accuracy by incorporating the CA125 biomarker.

## Results

### Patient baseline characteristics

From the initial cohort comprising 712 EOC patients receiving bevacizumab treatment, 127 individuals were omitted due to the unavailability of preprocessed CT plain images or suboptimal image quality, in addition to 60 patients with incomplete survival data. A comparison of baseline characteristics among the training set (*N* = 400), the internal test set (*N* = 97) and the external test set (*N* = 28) revealed no statistically significant differences, as depicted in Table 1.

**Table 1:** Comparison of baseline characteristics in training, internal and external test sets

Variable	Training set (*N* = 400)	Internal test set (*N* = 97)	External test set (*N* = 28)	*P*
Age				0.26
≤65 years	312 (78.0)	80 (82.5)	19 (67.9)	
>65 years	88 (22.0)	17 (17.5)	9 (32.1)	
BMI				0.35
<18	17 (4.3)	2 (2.1)	2 (7.1)	
18 ≤ X < 24	275 (68.8)	73 (75.3)	16 (57.1)	
24 ≤ X < 28	96 (23.5)	19 (19.6)	10 (35.8)	
≥28	12 (3.4)	3 (3.0)	0 (0.0)	
ECOG				0.29
0	97 (24.3)	19 (19.6)	13 (46.4)	
1	277 (69.3)	74 (76.3)	12 (42.9)	
2	26 (6.4)	4 (4.1)	3 (10.7)	
FIGO stage				0.33
Stage II	22 (5.5)	3 (3.1)	0 (0.0)	
Stage III	246 (61.5)	70 (72.2)	14 (50.0)	
Stage IV	132 (33.0)	24 (24.7)	14 (50.0)	
Pretreatment	382.5	592.3	301.0	0.17
CA125 level (U/ml)	(3.4–7410)	(5.9–7459)	(7.9–1287)	

### Performance of the ResNet18 DL model

**Fig. 1:**
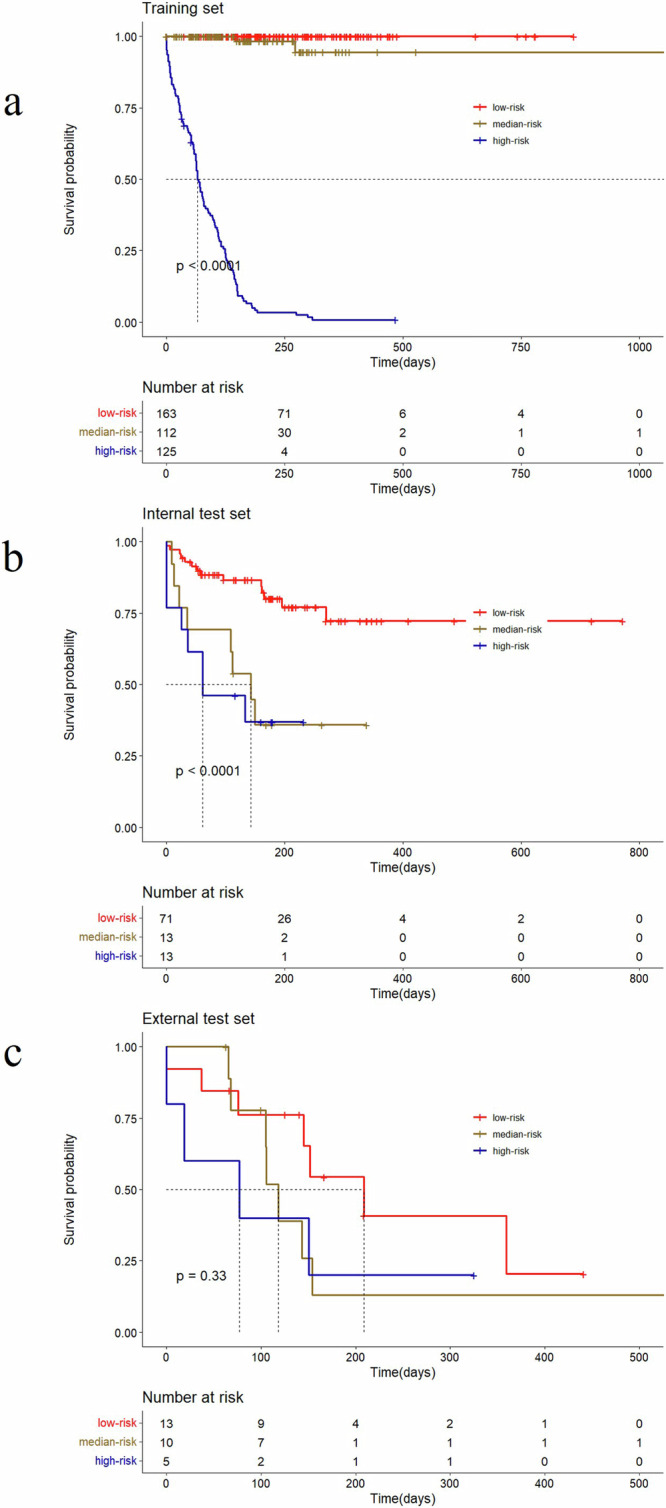
Kaplan–Meier curves showed ResNet18 DL model’s risk score. **a** Kaplan–Meier curves for PFS in training set; **b** Kaplan–Meier curves for PFS in internal test set; **c** Kaplan–Meier curves for PFS in external test set.

**Fig. 2:**
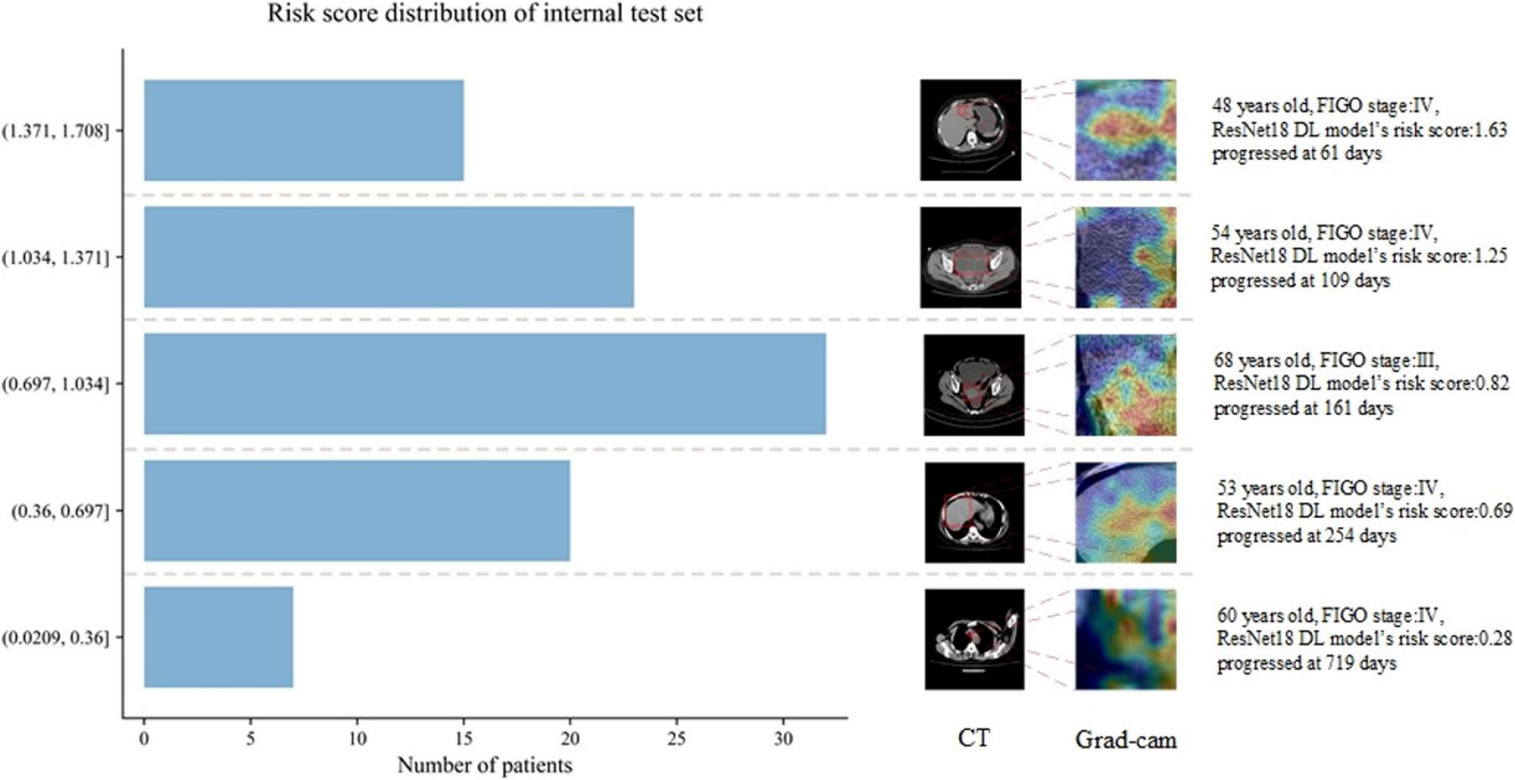
Histogram of the distribution of ResNet18 DL model’s risk score and examples of CT plain images. A histogram of the distribution of ResNet18 DL model’s risk score is shown on the left. Examples of CT plain images and the gradient-weighted class activation map (grad-cam) heat map are shown on the right.

A ResNet18 DL model was developed to predict PFS predicated on tumor volume segmented from preprocessed CT plain images^20^. The ResNet18 DL model achieved a C-index of 0.73 for predicting PFS in the internal test set and 0.61 in the external test set. Employing the training set, risk scores were computed based on the output of the ResNet18 DL model. Patients were then categorized into high-risk and low-risk groups utilizing the optimal cutoff value derived from the risk scores generated by the ResNet18 DL model. The Kaplan–Meier survival curves presented in Fig. 1 illustrate the survival outcomes of distinct patient groups, revealing a significant difference in survival probabilities between low-risk and median- and high-risk groups (*P* < 0.05). Nonetheless, the deep learning model exhibits limitations in accurately distinguishing between medium and high-risk groups. The clinical attributes characterizing these patient groups were outlined in Table 2. Furthermore, Fig. 2 showcased the distribution of risk scores calculated by the ResNet18 DL model and exemplars of original images within the internal test set.

**Table 2:** Characteristics of patients in high and low risk in training, internal and external test sets

Variable	Training set			*P*	Internal Test Set			*P*	External test set			*P*
	High risk (*N* = 125)	Median risk (*N* = 112)	Low risk (*N* = 163)		High risk (*N* = 13)	Median risk (*N* = 13)	Low risk (*N* = 71)		High risk (*N* = 5)	Median risk (*N* = 10)	Low risk (*N* = 13)	
Age				0.32				0.11				0.87
≤65 years	92	88	132		9	9	62		4	7	8	
	(73.6)	(78.6)	(81.0)		(69.2)	(69.2)	(87.3)		(80.0)	(70.0)	(61.5)	
>65 years	33	24	31		4	4	9		1	3	5	
	(26.4)	(21.4)	(19.0)		(30.8)	(30.8)	(12.7)		(20.0)	(30.0)	(38.5)	
BMI				0.06				0.51				0.95
<18	4	5	8		0	1	1		0	1	1	
	(3.2)	(4.5)	(4.9)		(0.0)	(7.6)	(1.4)		(0.0)	(10.0)	(7.7)	
18≤X<24	98	79	98		9	9	55		3	5	8	
	(78.4)	(70.5)	(60.1)		(69.2)	(69.2)	(77.5)		(60.0)	(50.0)	(61.5)	
24≤X<28	21	25	50		3	3	13		2	4	4	
	(16.8)	(22.3)	(30.7)		(23.2)	(23.2)	(18.3)		(40.0)	(40.0)	(30.8)	
≥28	2	3	7		1	0	2		0	0	0	
	(1.6)	(2.7)	(4.3)		(7.6)	(0.0)	(2.8)		(0.0)	(0.0)	(0.0)	
ECOG				0.09				0.33				1.00
0	31	26	40		4	3	12		2	5	6	
	(24.8)	(23.2)	(24.5)		(30.8)	(23.1)	(16.9)		(40.0)	(50.0)	(46.2)	
1	86	84	107		8	9	57		2	4	6	
	(68.8)	(75.0)	(65.6)		(61.6)	(69.3)	(80.3)		(40.0)	(40.0)	(46.2)	
2	8	2	16		1	1	2		1	1	1	
	(6.4)	(1.8)	(9.9)		(7.6)	(7.6)	(2.8)		(20.0)	(10.0)	(7.6)	
FIGO stage				0.88				0.20				0.30
Stage II	6	5	11		0	1	2		0	0	0	
	(4.8)	(4.5)	(6.7)		(0.0)	(7.6)	(2.8)		(0.0)	(0.0)	(0.0)	
Stage III	78	72	96		8	7	55		3	3	8	
	(62.4)	(64.3)	(58.9)		(61.5)	(53.9)	(77.5)		(60.0)	(30.0)	(61.5)	
Stage IV	41	35	56		5	5	14		2	7	5	
	(32.8)	(31.2)	(34.4)		(38.5)	(38.5)	(19.7)		(40.0)	(70.0)	(38.5)	
Pretreatment	384.4	378.4	382.6	0.99	612.5	508.9	486.7	0.68	317.4	286.9	302.6	0.53
CA125 Level (U/ml)	(3.4–7410)	(5.1–4724)	(4.3–5379)		(5.9–7459)	(7.1–4029)	(4.6–5076)		(9.8–1287)	(7.9–826.4)	(11.2–4682)

### Independent predictive ability of ResNet18 DL model

**Fig. 3:**
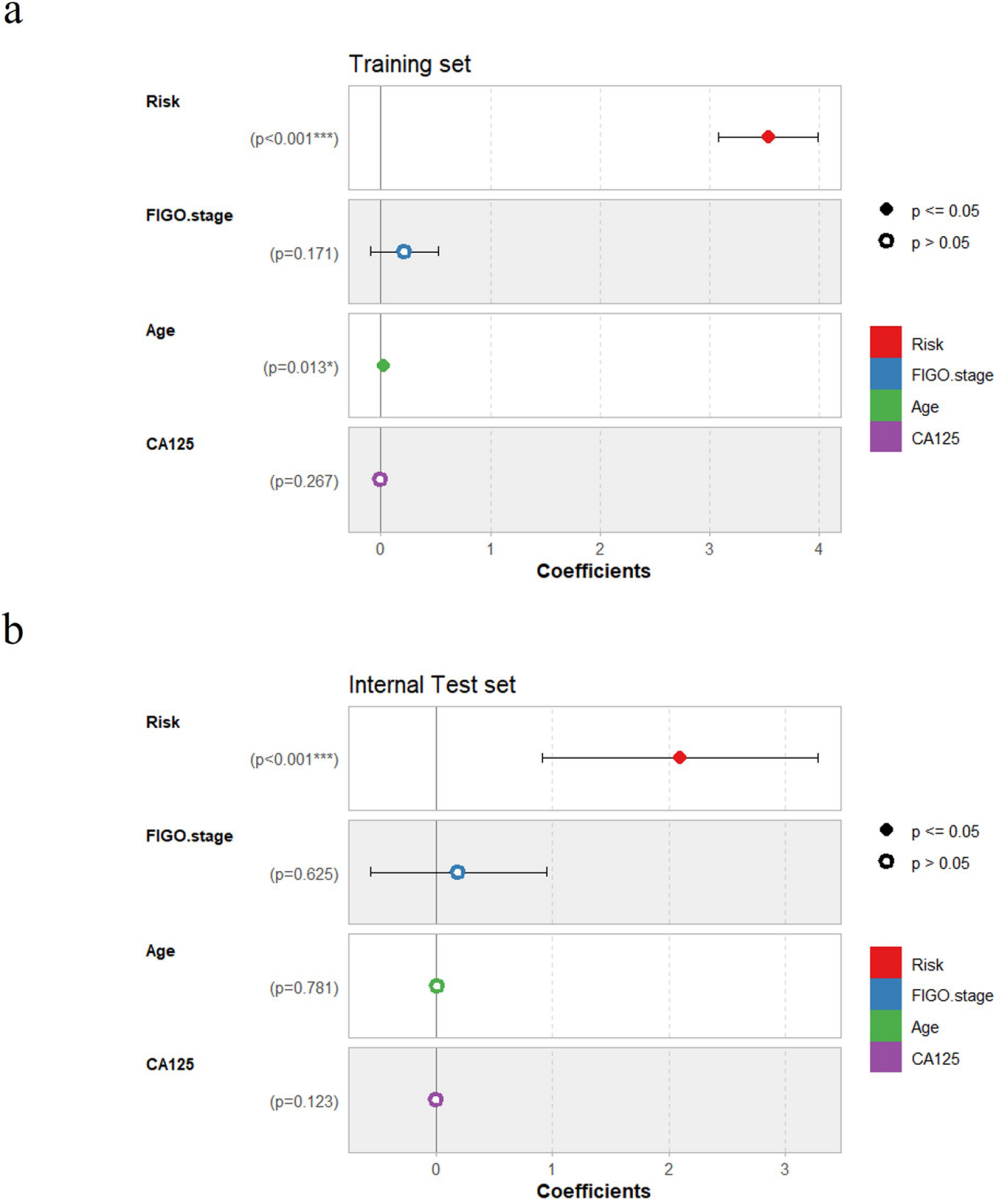
Forest plots for the multivariable Cox regression analysis. **a** Multivariable Cox regression analysis between ResNet18 DL model’s risk and clinical features for PFS in the training set; **b** Multivariable Cox regression analysis between ResNet18 DL model’s risk and clinical features for PFS in the internal test set. The multivariate Cox regression plot shows the coefficients representing the HR (Hazard Ratio) values for each variable. The horizontal lines indicate the 95% confidence interval for the HR values. Variables that have a significant impact are represented by solid symbols, while those considered not to have a significant impact are represented by hollow circles.

**Fig. 4:**
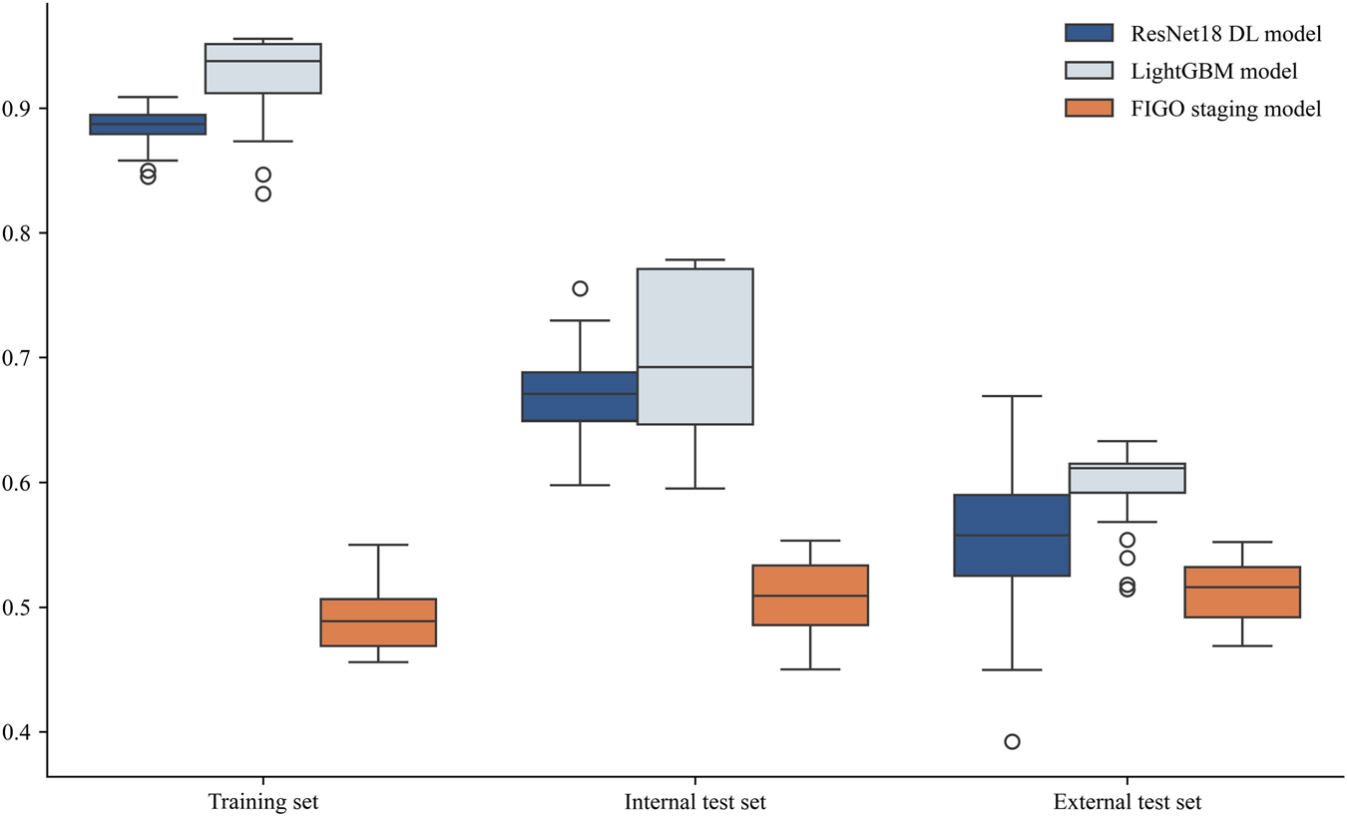
Comparison of Harrell C-indexes for LightGBM model, ResNet18 DL model and FIGO staging model. This boxplot compares the performance of three models—ResNet18 DL, LightGBM, and the FIGO staging model—across different datasets: training, internal test, and external test sets. Each boxplot shows the middle 50% of the data, with the top and bottom edges representing the third quartile (Q3) and first quartile (Q1), respectively. The box height (Q3 - Q1) indicates performance variability. The line inside the box shows the median (Q2), reflecting the central tendency. Whiskers extend from the box to 1.5 times the interquartile range, capturing typical values. Points outside this range are outliers, indicating extreme values that significantly deviate from the majority of the data.

As depicted in Fig. 3, regarding PFS, the multivariate Cox regression-adjusted hazard ratios (HR) pertaining to risk prediction derived from the ResNet18 DL model were determined to be 34.24 (95% CI: 21.7, 54.1; *P* < 0.001), 8.16 (95% CI: 2.5, 26.8; *P* < 0.001), surpassing those associated with FIGO stage. Analogously, comparisons of the C-index values for these variables also yielded consistent findings, as illustrated in Fig. 4.

### Performance of the LightGBM model

**Fig. 5:**
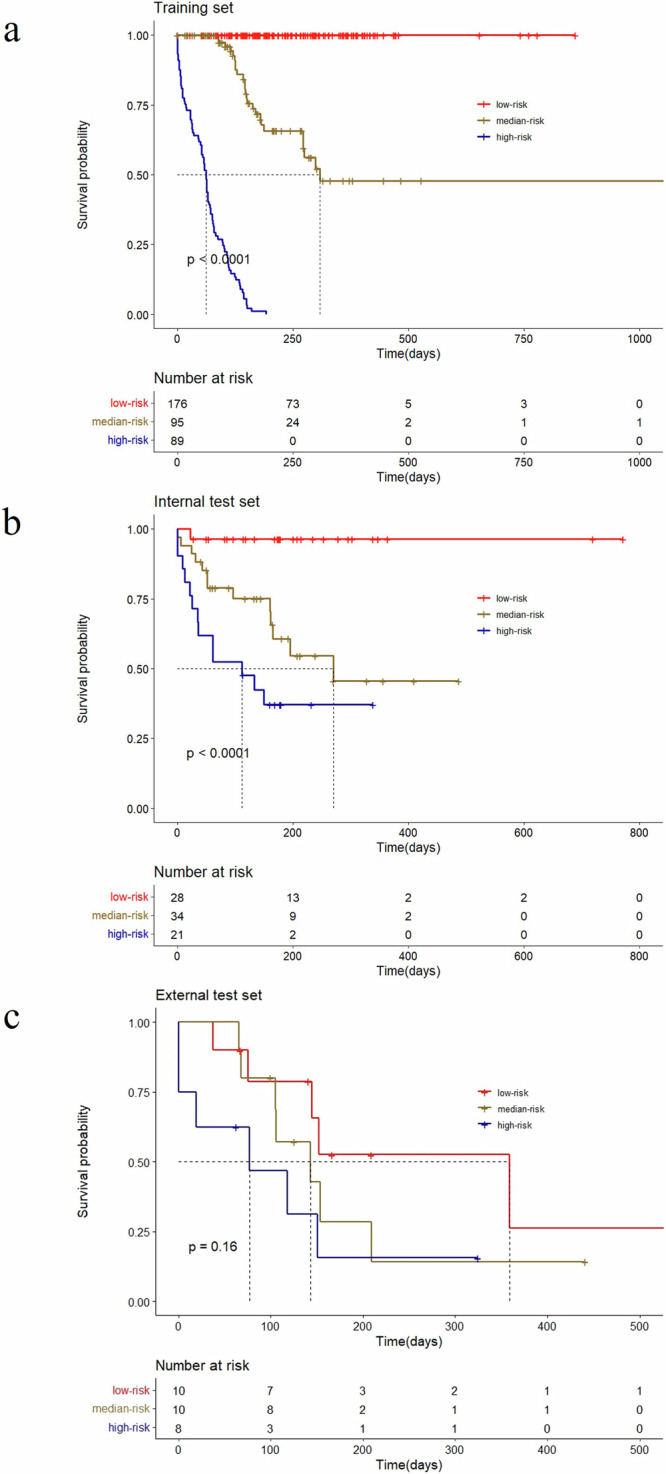
Kaplan–Meier curves showed LightGBM model’s risk score. **a** Kaplan–Meier curves for PFS in training set; **b** Kaplan–Meier curves for PFS in internal test set; **c** Kaplan–Meier curves for PFS in external test set.

The risk score was chosen as an input for the LightGBM model. Furthermore, we augmented the model by incorporating CA125, a pertinent tumor marker, which improved the predictive capacity for PFS among EOC patients receiving bevacizumab treatment (Fig. 4). The LightGBM model achieved a C-index of 0.76 for predicting PFS in the internal test set and 0.63 in the external test set. Elevated scores corresponded to heightened progression risk. Subsequently, the progression risk score was applied to the test set to validate its efficacy. The threshold value employed for stratifying the risk score aligns with the tertiles. Patients exhibiting elevated scores demonstrated a markedly escalated risk of progression compared to those with lower scores, as demonstrated in Fig. 5.

**Fig. 6:**
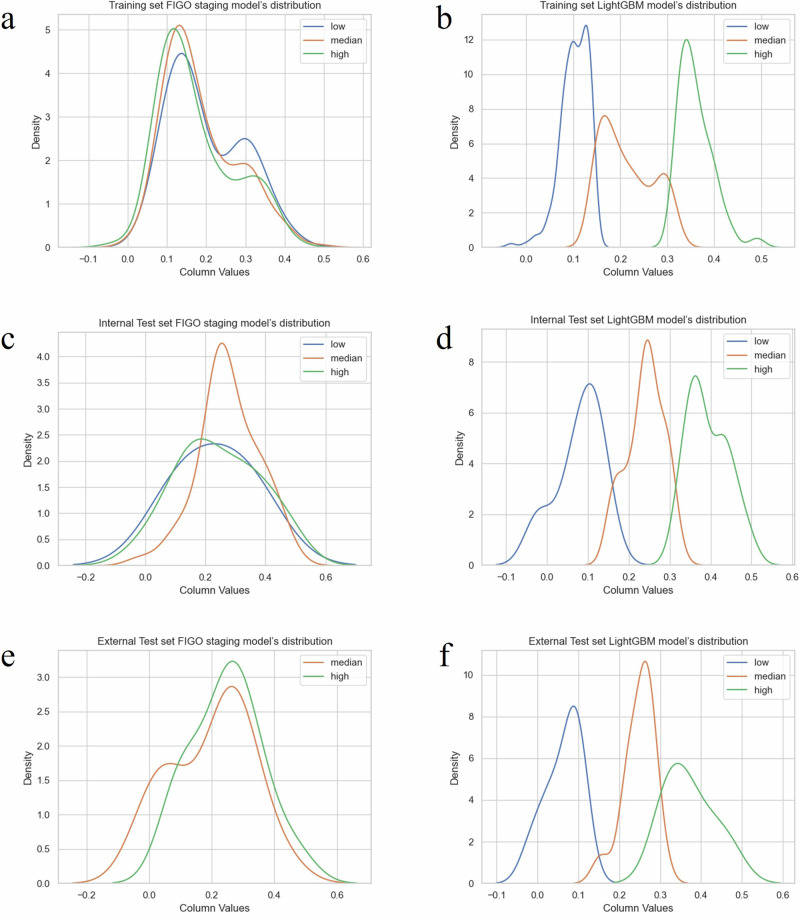
Discrimination and reclassification of patients by LightGBM model’s risk score. **a** Density plot of the FIGO staging model among training set; **b** Density plot of the LightGBM model among training set; **c** Density plot of the FIGO stage model among internal test set; **d** Density plot of the LightGBM model among internal test set; **e** Density plot of the FIGO stage model among external test set; **f** Density plot of the FIGO stage model among external test set. The horizontal axis represents the Risk Score values, while the vertical axis represents the corresponding population density. The integral of all probability density functions equals the total number of patients.

Figure 6 elucidated the delineation and reclassification of patients exhibiting adverse prognostic outcomes based on their respective scores. Notably, the scores assigned to high-risk progression patients markedly exceeded those assigned to their low-risk counterparts. As depicted in Table 3, comparison with the conventional classification relying on FIGO staging as a prognostic measure demonstrated the LightGBM model’s notable effect on prognostic reclassification. With the LightGBM model, grounded in the anticipated progression risk subsequent to bevacizumab treatment, the Net Reclassification Improvement (NRI) reached 0.06 (*P* < 0.001).

**Table 3:** Reclassification by the LightGBM model’s risk score of patients in overall cohort

Overall cohort	Low risk score	Median risk score	High risk score	NRI	*P*
FIGO staging model	21	325	169	0.06	<0.001
LightGBM model	103	215	197		

## Discussion

EOC represents a highly aggressive malignancy originating from ovarian tissues. The prognostication of EOC patients receiving bevacizumab treatment is contingent upon established indicators such as pretreatment FIGO stage^21,22^, disease recurrence, and treatment refractoriness, all of which significantly influence PFS outcomes. Extensive clinical investigations have underscored the considerable variability in PFS observed among EOC patients receiving bevacizumab treatment within both first- and second-line treatment contexts^5,23,24^. However, it is crucial to conduct additional research to determine the most effective timing and duration of bevacizumab treatment, as well as to evaluate its cost-effectiveness. Furthermore, identifying predictive markers that can differentiate between positive and negative treatment outcomes is an important area of focus. Utilizing these markers strategically has the potential to expand the range of therapeutic applications for bevacizumab and aid in selecting patient populations that will derive the greatest benefit from its use.

CT imaging plays a crucial role in modern medical practice by assisting in the development of treatment plans and the identification of medical conditions^25,26^. The extensive image data provided by CT scans allows for detailed analysis of various aspects of tumors in patients, such as the extent of metastasis, depth of infiltration, and spatial location^27–29^. This information is valuable for healthcare professionals in evaluating and predicting outcomes for cancer patients. Utilizing the capabilities of CT imaging, we utilized a ResNet18 deep learning model to predict PFS based on tumor volume extracted from preprocessed CT images. The ResNet18 DL model demonstrated significant predictive accuracy, as indicated by the multivariate Cox regression-adjusted hazard ratios obtained from the model. These hazard ratios were found to be 34.24 in the training set (95% CI: 21.7, 54.1; *P* < 0.001) and 8.16 in the internal test set (95% CI: 2.5, 26.8; *P* < 0.001), surpassing those associated with FIGO stage. Moreover, after accounting for relevant clinicopathologic variables such as FIGO stage, age, and CA125 levels, the risk score generated by the ResNet18 deep learning model remained a statistically significant independent predictor of patient outcomes. The subsequent stratification of patients into high-risk and low-risk groups, based on the optimal cutoff value obtained from risk scores, played a crucial role in identifying distinct survival trajectories. This was demonstrated by notable differences in survival probabilities between the two cohorts, as shown by Kaplan–Meier survival curves (*P* < 0.05). These results provide comprehensive validation of the prognostic utility of the ResNet18 deep learning model in informing treatment decisions for EOC patients receiving bevacizumab treatment.

Radiomics methodologies often require precise manual tumor annotation and empirical feature extraction^17,30,31^, which can limit the reproducibility and scalability of studies in this field. In contrast, our study employed a practical approach by carefully selecting the CT slice with the largest tumor region as the primary input for modeling. Additionally, in survival prediction research, accurately ordering survival time is crucial, unlike in image classification tasks. However, the existence of censored data within subsequent records presents a difficulty for utilizing ResNet18 deep learning models in predicting survival. Previous studies have often utilized binary classification methods to tackle this problem^32–34^. In our study, we implemented a customized loss function based on the Cox partial likelihood^35,36^ to address the varied range of survival risks among patients during the optimization process. This personalized loss function enabled continuous adjustments to the model parameters, with the primary goal of reducing overall loss. Additionally, we incorporated tailored loss functions related to PFS into the training dataset, allowing the ResNet18 deep learning model to capture the relevant characteristics linked to recurrence, metastasis, and mortality. This approach enhances the model’s predictive capabilities beyond individual event forecasts.

Identifying patients at high risk post-bevacizumab treatment completion continues to present a persistent challenge. The discriminative capabilities of the ResNet18 DL model’s risk score in distinguishing between high- and low-risk patients highlight its potential utility in identifying individuals at elevated risk who may benefit from more aggressive therapeutic interventions, even in cases where a favorable response is initially observed following bevacizumab treatment. Such integration of risk assessment scores could serve as a valuable adjunct to the decision-making process for these patients. The Harrell’s C-index serves as a crucial performance assessment measure for DL models, with a value of 1 indicating flawless model performance and a score of 0.5 or below suggesting inadequate performance on datasets. Therefore, a Harrell’s C-index approaching 1 indicates optimal training of the DL model. In the specific context of predicting PFS, the ResNet18 DL model achieved a Harrell’s C-index of 0.73 in the internal test set, demonstrating its noteworthy predictive accuracy.

This study introduced a LightGBM model, integrating CA125 and ResNet18 risk scores as inputs. CA125, a well-known blood biomarker, has been linked to unfavorable outcomes when found in elevated levels^37^. The Harrell’s C-index for the LightGBM model, in terms of predicting PFS, was calculated to be 0.76, outperforming the 0.73 achieved by the ResNet18 DL model alone. Furthermore, compared to the ResNet18 DL model, the LightGBM model can classify patients into more refined subgroups. We believe this is because while the ResNet18 DL model is trained solely on imaging data, the LightGBM model integrates both imaging and blood test features. Therefore, it can predict patient risk scores more accurately from the multimodal data, resulting in a higher c-index and evident differences in the KM curve outcomes. LightGBM builds upon the results of the ResNet18 DL model and incorporates blood test data, representing a progressive enhancement. Future research could investigate the inclusion of additional prognostic factors to further enhance the predictive accuracy of the model. In order to evaluate the effectiveness of the LightGBM model, the NRI metric was utilized to measure its ability to correctly reclassify patients compared to the established model. This metric aimed to assess how well the LightGBM model improved the classification of patient progression risk when compared to the traditional FIGO staging model. The results showed a significant NRI value of 0.06 (*P* < 0.001), indicating its substantial impact on prognostic reclassification when compared to the FIGO staging system.

The study is limited by factors such as its retrospective design. Additionally, the dataset used in this research may lack generalizability to populations in diverse geographic regions due to its reliance on data exclusively from the authors’ institution, potentially leading to variability in outcomes. To address these limitations, future research endeavors should prioritize external validation of the findings and incorporate data from a broader array of sources to enhance the robustness and applicability of the conclusions drawn from this study.

In conclusion, we developed and validated a DL model based on pretreatment CT imaging to prognosticate survival outcomes in EOC patients receiving bevacizumab treatment, obviating the need for manual feature extraction or selection. The risk score produced by our DL model demonstrates autonomous prognostic value and offers promise as a pretreatment risk assessment tool for the patients. While we have developed a model for EOC patients, our future research will focus on investigating deep learning prognostic methods for various subtypes of EOC. Additional validation through prospective clinical trials is necessary to confirm the effectiveness and consistency of our model in various clinical environments.

## Methods

### Data collection

This retrospective study was conducted in accordance with the ethical guidelines set forth in the Helsinki Declaration. Ethical approval was obtained from the Medical Ethics Committees of the First Affiliated Hospital of Anhui Medical University, the First Affiliated Hospital of Anhui Medical University High Branch and the Institutional Review Board of the Second People’s Hospital of Hefei. Given the retrospective design of the study, the need for informed consent was waived.

This retrospective study aimed to gather data on inoperable and recurrent EOC patients receiving bevacizumab treatment between January 2013 and January 2024 at three different medical institutions. Inclusion criteria comprised individuals meeting the following stipulations: (1) age ≥18 years; (2) histologically confirmed diagnosis of epithelial ovarian cancer; (3) FIGO stage II-IV classification; (4) Eastern Cooperative Oncology Group (ECOG) performance status ≤2; (5) absence of prior malignant neoplasms; (6) absence of concurrent severe chronic internal medical conditions; and (7) availability of clear and comprehensive CT imaging data acquired within a 2-week interval preceding the initiation of treatment.

**Fig. 7:**
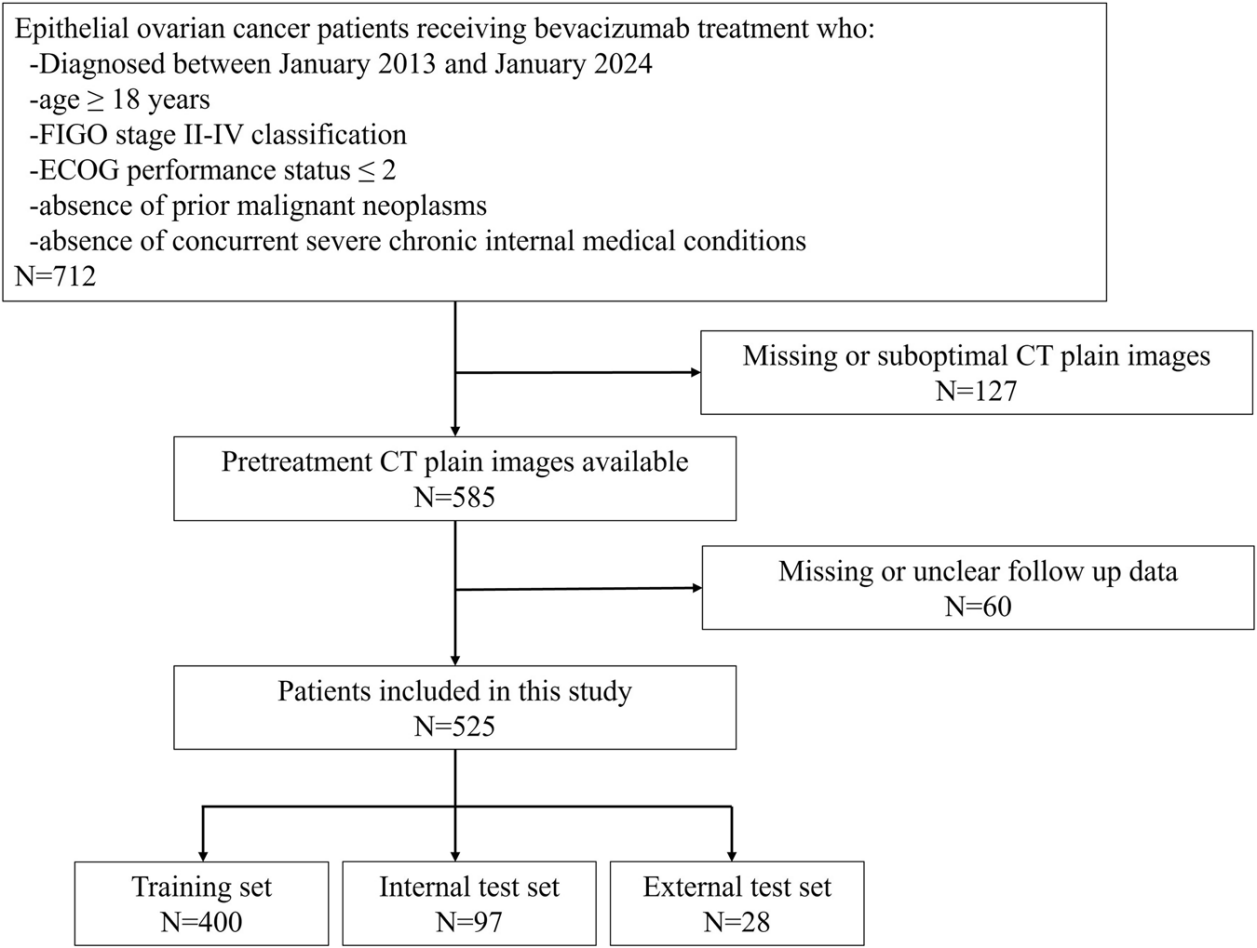
Screening of enrolled cases based on inclusion and exclusion criteria. Flowchart shows patients inclusion for the groups with epithelial ovarian cancer, which composed training set (*N* = 400), internal test set (*N* = 97) and external test set (*N* = 28).

**Fig. 8:**
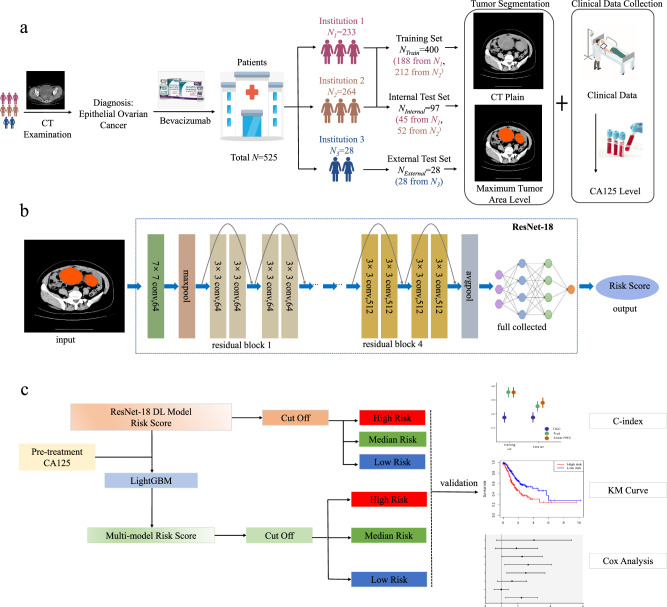
Workflow of this study. **a** Patient cohorts and data preprocessing. Workflow shows the development and validation of our model. **b** schematic diagram of the ResNet18 DL model. Diagram shows the model structure. **c** model validation. Flowchart shows the overall grouping and statistical analysis process, including the assessment of risk scores according to Kaplan-Meier (KM) survival analysis, Cox regression analysis and Harrell C-index.

Conversely, exclusion criteria were defined as follows: (1) age <18 years; (2) histologically confirmed non-EOC malignancies; (3) ECOG performance status >2; (4) concomitant presence of other malignancies; (5) concurrent severe chronic internal medical conditions; and (6) discernible artifacts, blurring, errors, or disordered slices evident within CT imaging. Within the initial group of 712 EOC patients receiving bevacizumab treatment, 127 individuals were eliminated from the study due to the absence of preprocessed CT plain images or suboptimal image quality, in addition to 60 patients with incomplete survival data. As a result, a total of 525 EOC patients from the designated institutions were included in the study, with 484 patients diagnosed with high-grade serous carcinoma, 21 patients with low-grade serous carcinoma, and 20 patients with clear cell carcinoma. Prior to the initiation of treatment, baseline assessments, including physical examinations, tumor marker evaluations, and CT scans, were carried out for all participants. Data collection for this investigation persisted until January 2024. The patient cohort for this study was visually represented in Figs. 7 and 8a, with a total of 400 cases from institutions 1 and 2 included in the training set, 97 cases from institutions 1 and 2 included in the internal test set, and 28 cases from institution 3 included in the external test set.

### Patient cohorts

A total of 525 EOC patients receiving bevacizumab treatment between January 2013 and January 2024 were retrospectively analyzed. As shown in Fig. 8a, regular monitoring of tumor markers was implemented during the treatment phase, with follow-up assessments conducted within 2 months post-treatment culmination. During these follow-up evaluations, comprehensive blood analyses including routine hematology, biochemical profiling, and tumor marker assessments were performed. Furthermore, efficacy evaluation entailed physical examinations and computed tomography (CT) scanning, adhering to the Response Evaluation Criteria in Solid Tumors (RECIST) guidelines (version 1.1)^38^. Tumor response on CT scans was adjudicated based on the vertical length and maximum transverse thickness of the lesion. Complete response (CR) was characterized by the absence of discernible primary tumor areas, while partial response (PR) was defined as a reduction of ≥30% in the sum of diameters of all measurable target lesions, sustained for a minimum of 4 weeks relative to baseline measurements. Progressive disease (PD) was delineated by either a ≥20% increase from baseline in the sum of target lesion diameters, an absolute increase of ≥5 mm, or the emergence of new lesions. Stable disease (SD) was identified by fluctuations in lesion volume and number, exhibiting characteristics between partial response and disease progression. Post-treatment, follow-up appointments were scheduled bi-monthly in the initial year, tri-monthly in the subsequent two years, and semi-annually thereafter. Disease progression was ascertained utilizing RECIST criteria, incorporating clinical manifestations, imaging modalities, or escalating levels of CA125^39^.

### Data preprocessing

The study systematically documented patient demographics, including variables such as age, height, weight, body mass index (BMI), ECOG performance status, pre-treatment FIGO stage, and pre-treatment blood tumor markers, as clinical attributes. The target variable of interest was PFS data. CT plain imaging data acquired prior to treatment initiation were retained for analysis. Two experienced clinicians, each with more than ten years of clinical experience, manually outlined the boundaries of the tumor on each CT slice corresponding to the EOC tumor region. Following this, a senior clinician with over twenty years of expertise reviewed and modified these outlines. For original DICOM images, we apply a windowing technique using Window Width (WW) and Window Level (WL) parameters tailored to the mediastinum window. Grayscale processed CT images undergo min-max normalization to scale pixel values to 0-1 range, followed by histogram equalization with a contrast threshold of 2.0 and a grid size of 8 × 8. This is succeeded by another round of min-max normalization before inputting data into the network. Regarding CA125 data from blood tests, values like ‘>1000’ and ‘>500’ are replaced with their respective numeric values of 1000 and 500. During model training, min-max normalization is applied to CA125 values across all cases, mapping them to the 0-1 range. The CT slice containing the largest tumor region was selected as the input for further modeling efforts (Fig. 8a).

### DL model construction

A deep learning architecture, specifically Residual Network (ResNet18)^40^ pretrained on imagenet-1k (ImageNet: A large-scale hierarchical image database) was developed to predict PFS predicated on tumor volume segmented from preprocessed CT plain images. Patients from two different institutions were randomly divided into training and internal test subsets at an 8:2 ratio. Among them, 188 patients from institution one and 212 patients from institution two were assigned to the training set, while 45 patients from institution one and 52 patients from institution two were assigned to the internal test set (Fig. 8a). Patients from the third institution were gathered to form an external test set. The study involved conducting comparative experiments using pre-trained deep learning models, including ResNet18, ResNet50, DenseNet, and ViT, all trained on ImageNet-1K. Each experiment was repeated ten times for each model, with the model parameters saved for analysis. The ResNet18 deep learning model with the highest overall C-index was selected for further analysis. This model was then used to develop a predictive model aimed at estimating patient risk scores directly from preprocessed CT plain images depicting segmented tumors (Fig. 8b). These images encompassed a region of interest (ROI) centered on the tumor, along with a 3-pixel margin surrounding the tumor’s periphery. During the optimization phase, a customized loss function based on the Cox partial likelihood^41^ was utilized to address the diverse levels of survival risk among patients. This loss function enabled iterative adjustments to the model parameters with the goal of reducing overall loss. Figure 8b illustrated a schematic representation of the ResNet18 deep learning model for clarification.

### Statistical analysis

The primary methodologies utilized for assessing the prognostic efficacy of the developed model were Harrell’s C-index and Kaplan–Meier survival analysis. Patients were categorized into different risk groups based on disease progression, and Kaplan–Meier survival curves were generated for each group. Disparities in survival prognosis among these groups were quantified through the calculation of *P* values, where *P* < 0.05 indicates a significant difference in survival prognosis. Kaplan–Meier survival analysis was employed to examine prognostic differences between these risk groups. Furthermore, post-hoc analyses were undertaken to discern potential associations between variables such as age, FIGO stage, CA125 levels, and various patient groups and subgroups.

As shown in Fig. 8c, a light gradient boosting machine (LightGBM)^42^ learning model was developed using CA125 and ResNet18 risk scores as input variables. The LightGBM model was further processed with weights to integrate multimodal data and derive a progression risk score tailored to EOC patients receiving bevacizumab treatment. Subsequently, patients were categorized into different risk groups based on disease progression, and Kaplan–Meier survival curves were generated for each group. Disparities in survival prognosis among these groups were quantified through the calculation of *P* values, where *P* < 0.05 indicates a significant difference in survival prognosis.

The comparative analysis of these models included an evaluation of the C-index of the ResNet18 DL model, the LightGBM model, and the conventional FIGO staging model, each utilized independently for prognosis prediction. This evaluation was enhanced by the incorporation of the Net Reclassification Improvement (NRI) metric^43^, which measured the extent to which a new model correctly or incorrectly reclassified patients compared to an existing model. The overarching objective was to assess the degree to which the model with the highest C-index in the internal test set improved the classification of patient progression risk compared to the traditional FIGO staging model. A significance threshold of *p* < 0.05 was employed to determine if the new model significantly enhanced the accuracy of patient progression risk classification.

Statistical analyses were performed using R 3.4.0 software. Continuous variables were assessed using t-tests, while categorical variables were analyzed using either the χ2 test or Fisher’s exact test, as deemed appropriate. The primary endpoint of interest in this study was PFS, defined as the duration from the initiation of bevacizumab treatment to either tumor progression or tumor-related death, or until the date of the last follow-up. All statistical tests were two-tailed, with significance set at a threshold of *p* < 0.05.

## Supplementary information

supplementary information

## Data Availability

All data generated or analyzed during this study are included in this article. Further inquiries can be directed to the corresponding authors.

## Code availability

The code can be used only for non-commercial purpose and under the permission of the corresponding authors. Source code used in this study can be found at https://github.com/phaeton2017/PredictionEOC.
