# Supplementary material for: Predicting prognosis for epithelial ovarian cancer patients receiving bevacizumab treatment with CT-based deep learning
Source: NPJ Precis Oncol. 2024 Sep 13;8:202. doi: 10.1038/s41698-024-00688-6 (PMC11399346; doi:10.1038/s41698-024-00688-6)
Supplement: Supplementary file 1 — supplementary information [file 41698_2024_688_MOESM1_ESM.pdf]

Supplementary Information

Supplementary Tables:

Supplementary Table 1. Comparison of Model Performances between VIT, DenseNet, ResNet50 and ResNet18 based on the consistency index evaluation criterion. The table below shows the mean and the 95% confidence interval for the training set, internal test set, and external test set.

Supplementary Table 2. Comparison of Model Performances between VIT, DenseNet, ResNet50 and ResNet18 based on the training time, memory usage and floating-point operations per second (flops).

Supplementary Table 3. Preprocessing for the original DICOM images

Supplementary Table 4. The mean and 95% confidence intervals of the consistency index on the split datasets

Supplementary Figures:

Supplementary Figure1. The consistency index interval plot

Supplementary Figure2. Illustrative examples clarifying the largest tumor segmentation process

Supplementary Figure3. The interval plot based on the mean and 95% confidence intervals of the consistency index on the split datasets

Supplementary Tables

Supplementary Table 1 Comparison of Model Performances between VIT, DenseNet, ResNet50 and ResNet18 based on the consistency index evaluation criterion. The table below shows the mean and the 95% confidence interval for the training set, internal test set, and external test set.

METHOD	Training set	Internal test set	External test set
VIT	0.830(0.771, 0.888)	0.651(0.621, 0.680)	0.537(0.483, 0.591)
DenseNet	0.871(0.847, 0.896)	0.662(0.637, 0.687)	**0.570(0.538, 0.602)**
ResNet50	**0.884(0.852, 0.916)**	0.676(0.627, 0.725)	0.548(0.496, 0.600)
ResNet18	0.863(0.821, 0.906)	**0.677(0.630, 0.725)**	0.564(0.484, 0.643)

Supplementary Table 2 Comparison of Model Performances between VIT, DenseNet, ResNet50 and ResNet18 based on the training time, memory usage and floating-point operations per second (flops).

METHOD	Params(M)	Flops(GFlops)	Training time(s)
VIT	85.6	16.86	4.22
DenseNet	6.95	2.89	2.60
ResNet50	23.5	4.13	2.23
ResNet18	**11.2**	**1.8**	**0.77**

Supplementary Table 3 Preprocessing for the original DICOM images

Algorithm : Windowing Technique for Grayscale

Input: CT image, window level WL, window width WW

Output: CT image with windowing technique applied new_image

Algorithm steps:

1. Calculate the minimum HU value: $minHU \leftarrow WL - 0.5 * WW$
2. Window leveling: $new\_image \leftarrow (image - minHU)/WW$
3. Window clipping: $new\_image[new\_image \leq 0] = 0$
   $new\_image[new\_image \leq 1] = 1$
4. Map to the range [0, 255]: $new\_image = new\_image * 255$

Supplementary Table 4 The mean and 95% confidence intervals of the consistency index on the split datasets

METHOD	Training set	Internal test set	External test set
FIGO staging model	0.489(0.455, 0.524)	0.509(0.463, 0.554)	0.510(0.468, 0.553)
ResNet18 DL model	0.863(0.821, 0.906)	0.677(0.630, 0.725)	0.564(0.484, 0.643)
LightGBM model	**0.921(0.882, 0.961)**	**0.732(0.662, 0.802)**	**0.589(0.533, 0.645)**

Supplementary Figures

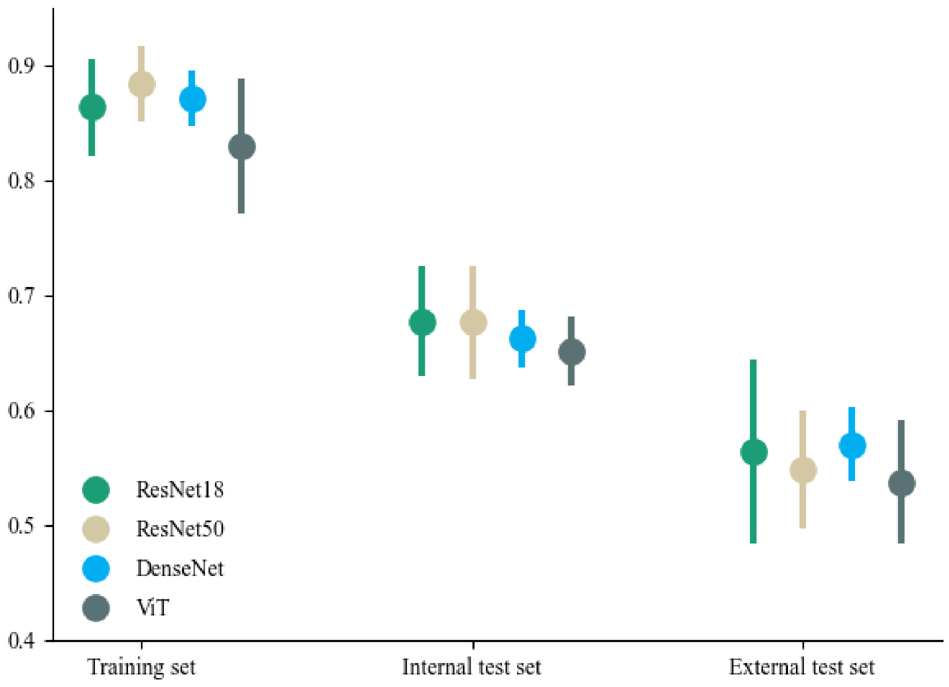

Supplementary Figure 1 The consistency index interval plot

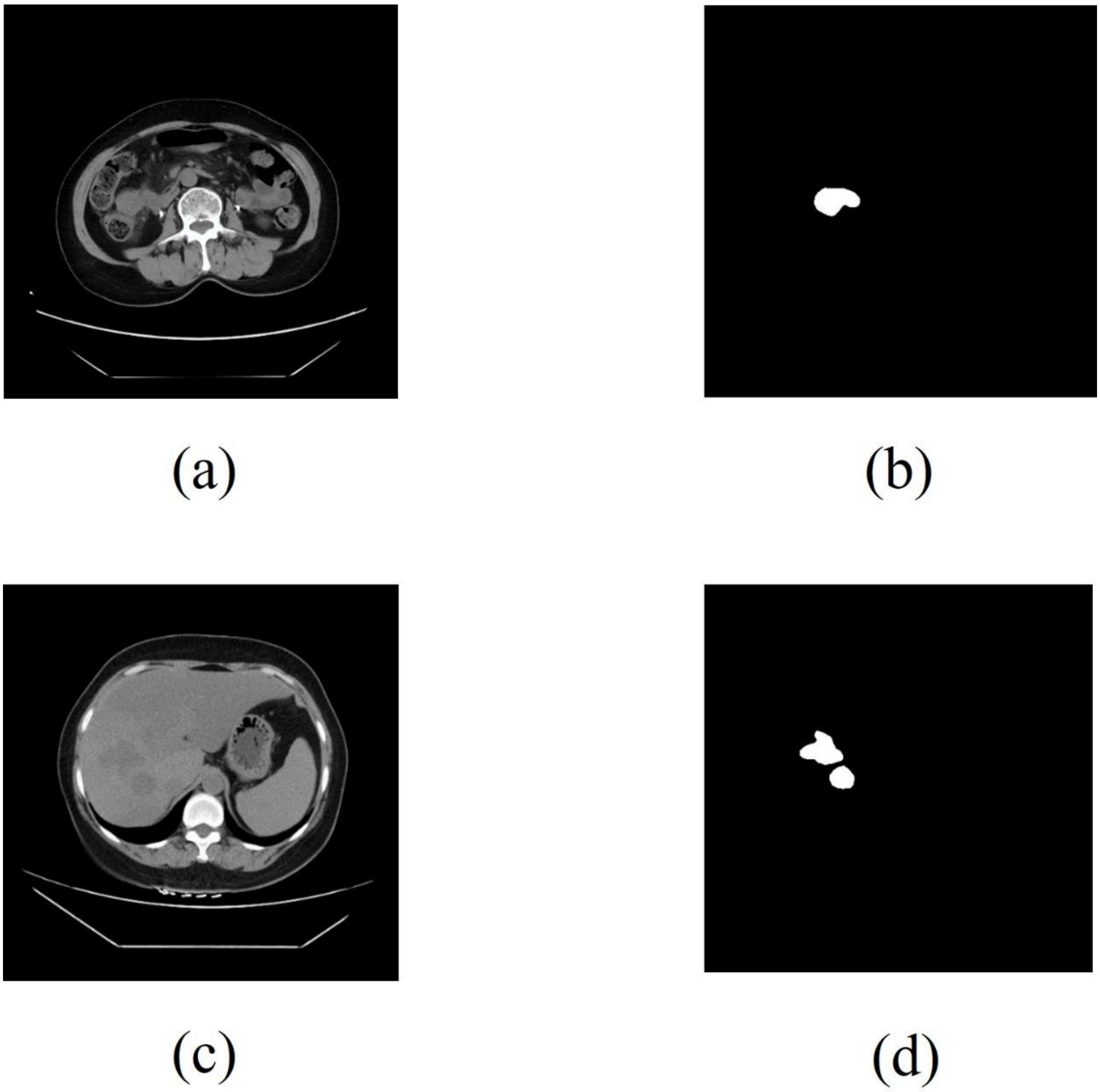

Supplementary Figure 2 Illustrative examples clarifying the largest tumor segmentation process

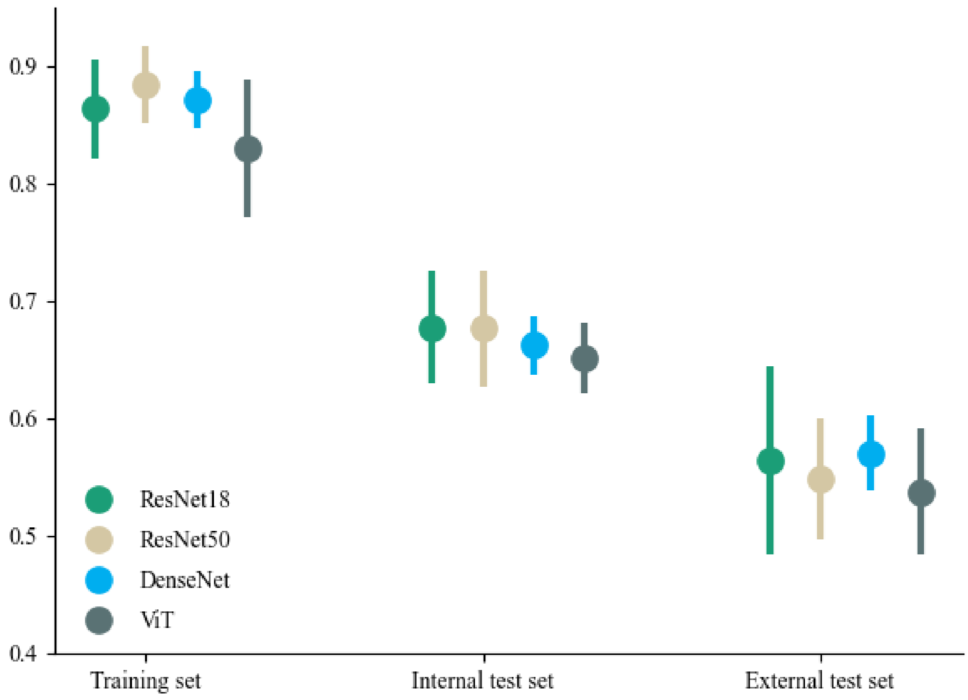

Supplementary Figure 3 The interval plot based on the mean and 95% confidence intervals of the consistency index on the split datasets
